# The correlation between caregiver burden with depression and quality of life among informal caregivers of hemodialysis and thalassemia patients during the COVID-19 pandemic: a cross-sectional study

**DOI:** 10.1186/s12912-023-01351-4

**Published:** 2023-05-29

**Authors:** Maryam Askaryzadeh Mahani, Masoomeh Ghasemi, Mansour Arab, Zahra Baniasadi, Ali Omidi, Parichehr Sabaghzadeh Irani

**Affiliations:** 1 Student Research Committee, Razi faculty of Nursing and Midwifery, Kerman University of Medical Sciences, Kerman, Iran; 2 Bam University of Medical Sciences, Bam, Iran; 3 School of Nursing ad Midwifery, Shahid Beheshti University of Medical Sciences, Tehran, Iran

**Keywords:** Caregiver burden, Thalassemia, Hemodialysis, Depression, Quality of life

## Abstract

**Background:**

Lifelong provision of care to chronically ill patients increase the risk of physical and mental diseases in informal caregivers and adversely affects their quality of life. The present study examined the correlation between caregiver burden, depression, and quality of life among the informal caregivers of thalassemia and hemodialysis patients during the COVID-19 pandemic in southeastern Iran.

**Methods:**

This cross-sectional correlational study used convenience sampling to select 200 informal caregivers involved in providing direct care for patients undergoing hemodialysis (n = 70) and patients with thalassemia (130) for at least 6 months. A demographic questionnaire, Beck’s Depression Inventory (BDI), the Quality-Of-Life Questionnaire (SF-36), and the Zarit Burden Interview were used to collect data in 2021. The data were analyzed with SPSS software (version 19) using frequency, percentage, independent samples t-test, ANOVA, and multivariate regression analysis.

**Results:**

Most of the informal caregivers of the thalassemia and hemodialysis patients (58% and 43%) reported moderate levels of caregiver burden. There were significant correlations between the caregiver burden and depression (P < 0.0001) and between the caregiver burden and the quality of life (P < 0.009). The level of depression in informal caregivers of patients undergoing hemodialysis was higher than that of the informal caregivers of patients with thalassemia, but the quality of life in the informal caregivers of the patient’s undergoing hemodialysis was higher than that of the informal caregivers of the patients with thalassemia.

**Conclusion:**

Considering the significant correlations between caregiver burden, depression, and quality of life in this study, healthcare providers are recommended to develop educational and supportive interventions to meet informal caregivers’ needs, mitigate their emotional distress, fears, and concerns, and prevent caregiver burden in times of greater uncertainty.

## Background

The prevalence of chronic diseases is increasing in the world so they can affect family functioning [1], [2]. Given the traditional norms of families in Asian countries, some members of these families play the role of informal caregivers [3]. The term “informal caregiver” refers to an unpaid family member, friend, or neighbor who provides free care to a sick individual [4, 5]. Some of the informal caregivers’ responsibilities are to assist patients with their personal affairs, give them medicines, transfer them to the medical center, help them eat, and support them emotionally and psychologically [6]. Informal caregivers of chronically ill patients may experience extensive levels of caregiver burden since they have an important role in supporting these patients [7].

Caregiver burden is perceived by the caregiver while caring for his/her family member and/or a loved one over time [8]. It is a multidimensional response to physical, psychological, emotional, social, and financial stressors [9]. Caregivers are hidden patients who may not be able or eager to seek care for their own health needs [10]. Disruption in the caregiver’s daily activities, recreation, and social communication as well as the patient’s disability and disease progress can be some consequences of caregiver burden [2].

Given that chronic diseases change a person’s life, the diversity and severity of caregiving roles can lead to psychological problems in the informal caregivers of patients [11]. Caregivers’ mental health can be even more at risk when the patient’s care needs exceed their caregiving capabilities [12]. An increase in caregiver burden among informal caregivers may have different consequences, such as family isolation, loss of hope for social support, disruption in family relationships, inadequate care of the patient, and ultimately abandonment of the patient [13]. Informal caregivers may feel insecure, uncomfortable, exhausted, anxious, and depressed [2].

COVID-19 or severe acute respiratory syndrome coronavirus 2 (SARS-CoV-2) was first discovered in Wuhan, China in December 2011 [14]. The pervasiveness of COVID-19 turned into a major health problem worldwide. On March 11, 2020, the World Health Organization declared the COVID-19 outbreak a global pandemic [15]. The way the disease is transmitted, the news that is brought to the public in various ways, and the risk of mortality caused by the virus can negatively affect people’s mental health [16].

Measures taken to control the COVID-19 pandemic, including isolation and quarantine, lockdowns, cancellation of flights, evacuation of foreign people, closures of schools and universities, etc., can intensify these fears [17]. Bendau reported that individuals’ psychological stress increased through social distancing measures [18].

Patients receiving hemodialysis require regular treatment sessions and have to use public transportation several times a week, and they are at a greater risk of infectious diseases due to their weak immune systems, aging, and co-morbidities [19]. Patients with thalassemia need regular blood transfusions and proper follow-ups [20], but we witnessed a significant decrease in voluntary blood donation with the onset of the COVID-19 pandemic. These problems also affect the informal caregivers’ mental health and quality of life [21]. Quality of life is the physical, social, and psychological aspects of well-being and it is affected by the COVID-19 disease and its treatment [22]. The sudden transmission of the novel coronavirus along with instant measures taken in response to the Coronavirus Disease 2019 (COVID-19) pandemic caused many new challenges adversely disturbed the quality of life [21]. For instance, Pasteur Hospital located in Bam, Iran applied strict health protocols during the pandemic, so the number of patient companions was limited during hemodialysis sessions, and every patient undergoing hemodialysis had to get the COVID-19 swap test 24 h before hemodialysis. This policy indirectly created a separate burden on caregivers of hemodialysis patients because they had to spend some money for a COVID-19 swap test at least 2 times a week.

Patients with thalassemia major had a serious dilemma of whether to stay at home and postpone blood transfusions, which could increase the risk of severe anemia and iron overload. The process of caring for this patient could cause a caregiver burden on the informal caregivers. Thus, informal caregivers are vulnerable to such stressors because the patient’s biological, social, and psychological demands overwhelm their own needs [23].

Studies on the informal caregivers of thalassemia and hemodialysis patients showed conflicting results. Some of these studies reported moderate to severe levels of caregiver burden among the informal caregivers of these patients [23–25], while others reported a low caregiver burden [6]. The informal caregivers’ psychological condition and quality of life can undergo many changes. Previous qualitative studies have suggested that the provision of care during the lockdown was even more challenging, as informal caregivers faced greater emotional strains due to increased care responsibilities. However, the impacts of the COVID-19 pandemic on these informal caregivers remain largely unexplored [26].

Determining the level of caregiver burden, planning a program to reduce this burden, as well as examining the correlation between caregiver burden, depression, and quality of life of the informal caregivers of thalassemia and hemodialysis patients can play an important role in improving the general condition of informal caregivers and their quality of care. This study aimed to determine the correlation between caregiving burden, depression, and quality of life in the informal caregivers of thalassemia and hemodialysis patients during the COVID-19 pandemic.

## Methods

### Design

This cross-sectional correlational study was conducted on the informal caregivers of the thalassemia and hemodialysis patients admitted to Pasteur Hospital in Bam during the COVID-19 pandemic in July 2021.

### Study setting and sample

The study population comprised informal family caregivers of the thalassemia and hemodialysis patients, who had medical records in Pasteur Hospital in Bam. The total number of.

patients admitted to this hospital were 200 (70 patients undergoing hemodialysis and 130 patients with thalassemia). The participants were selected using convenience sampling. The main inclusion criterion was the patient’s dependency on care which was assessed by two questions about the patient’s ability to do his/her daily activities and his/her need for care. The informal caregivers of those patients who could do their daily activities were excluded from the study. A family relationship between the patient and the caregiver, the patient and caregiver’s willingness to participate in the study, the caregiver’s direct involvement in the patient’s care, suffering from the disease for at least 6 months (patient), age between 18 and 65 years (caregiver), no payment for caring, and no history of chronic mental illness (in the caregiver) were other inclusion criteria in this study. No participants refused to participate or withdrew after giving their consent.

Bam is a city located in Kerman Province, southeastern Iran. There is a public hospital and a private hospital affiliated with Bam University of Medical Sciences. Since the thalassemia and hemodialysis departments are located only in the public hospital, so all the patients admitted to this hospital were included in this study based on census sampling. Therefore, we used no formula in the present study to estimate the sample size.

### Data collection

The data were collected using a demographic questionnaire, Beck’s Depression Inventory, the Quality-Of-Life Questionnaire (SF-36), and the Zarit Burden Interview. Demographic information included the caregiver’s age, sex, marital status, education, income, the effect of Covid-19 on patient conditions, and relationship with the patient.

### Beck’s depression inventory

The BDI is a 21-item self-report instrument that measures depression severity. All items in the BDI are rated on a four-point Likert scale ranging from 0 to 3, and the total score ranges from 0 to 63 [27, 28]. Beck et al. developed the revised version of the BDI to match (BDI-II) contents with the modern diagnostic criteria for MDD set out in the Diagnostic and Statistical Manual of Mental Disorders (DSM)-IV while maintaining the same number of items and range of scale as the BDI [29]. Scores 0–13 indicate minimal depression, 14–19 indicate mild, 20–28 indicate moderate, and 29–63 indicate severe depression [30]. The BDI has sufficient internal consistency in psychiatric patients (Cronbach’s *α* ranging from 0.76 to 0.95) and non-psychiatric populations (Cronbach’s *α* ranging from 0 0.73 to 0.92). The BDI-II also has acceptable internal consistency among college students (*α* = 0.93) and outpatients *(α* = 0.92). According to a survey of 1022 undergraduate students, the mean score of the BDI-II was 1.54 points higher than that of the BDI, but the two scales showed a high correlation (*r* = 0.93), suggesting good convergence of the two scales [27, 31].

### Quality of life questionnaire (SF-36)

The Quality-of-Life Questionnaire (Varosherbon,1992) contains 36 items measuring eight dimensions of general health, physical functioning, physical pain, social functioning, vitality, mental health, role restriction due to physical problems, and role restriction due to mental problems. Each dimension gains a score from 0 to 100, with higher scores indicating better quality of life. Scores 71–100 indicate desirable, 31–70 indicate somewhat desirable, and 0–30 indicate undesirable qualities of life. This questionnaire was designed to measure health-related quality of life, and the reliability and validity of its Iranian version were confirmed [32]. The quality-of-life questionnaire (SF-36) is a standard instrument for use in clinical research, evaluating health policy, and public health. Barazir et al. in the UK (1992) reported that its reliability was more than 85% using Cronbach’s alpha test [33]. Montazeri et al. in Iran translated and determined the reliability and validity of the Persian version of the SF-36 questionnaire. They showed that, except vitality dimension (α = 0.65), other dimensions gained minimum standard reliability coefficients (ranging from 0.77 to 0.9) [34].

### Zarit burden interview

The scale was developed by Zarit et al. (1998) to measure caregiver burden and contains 22 items measuring personal, social, emotional, and economic burdens. The items are scored on a 5-point Likert scale (never = 0, 1 = rarely, 2 = sometimes, 3 = often, and 4 = nearly always). The total scores vary from 0 to 88, with the lower score indicating less caregiver burden. Scores 0–20 indicate low or lack of caregiver burden, 21–40 indicate moderate caregiver burden and 41–88 indicate severe caregiver burden [35]. Shafiezadeh confirmed the face and construct validity and internal consistency of this scale [36]. The researcher asked the informal caregivers questions in person, but if they wanted to complete the questionnaire, she gave them the questionnaire and then collected it.

### Ethical considerations

The Ethics Committee of Kerman University of Medical Sciences approved the protocol for this study on June 5, 2021. After obtaining the necessary permits and making arrangements with the relevant authorities, the researcher received written informed consent from the participants. She also assured them that their information would be kept confidential and they would not experience any physical or moral harm, and then informal caregivers completed the items in the questionnaires within 30–40 min in the research settings.

### Statistical analyses

Before the analysis, the normal distribution of the variables was assessed using the Kolmogorov–Smirnov Test (p > 0.05). Data analyses were conducted using SPSS 19. Mean, standard deviation, frequency, and percentage were used for descriptive statistics, while independent t-test, ANOVA, and Pearson correlation coefficient were used for analytical statistics. A significant level of P ≤ 0.05 was considered. A multiple regression analysis was performed to identify potential predictors of caregiver burden during the COVID-19 pandemic.

## Results

Among 200 participants studied, 56% were male and 47% were single, 65% of the caregivers were caring for patients with thalassemia and 35% were caring for patients undergoing hemodialysis. Moreover, 78.5% of the informal caregivers reported that the COVID-19 pandemic worsened the patient’s condition. The mean age of the participants was 31.37 ± 22.03 years. Table 1 depicts the participants’ descriptive characteristics.

**Table 1:** The participants’ demographic characteristics

Variable		Frequency	Percentage (%)
**Age**	30<	101	50.5
	30–60	86	43
	60>	13	6.5
**Sex**	Male	112	56
	Female	88	44
**Marital status**	Married	106	53
	Single	94	47
**Education**	Masters	15	7.5
	Associate degree	35	17.5
	Elementary	101	50.5
	Illiterate	49	24.5
**Ward**	Hemodialysis	70	35
	Thalassemia	130	65
**Job**	Unemployed	56	28
	Self-employed	40	20
	Housewife	36	18
	Student	54	27
	Employed	14	7
**Financial situation**	Weak	32	16
	Medium	125	62.5
	Good	43	21.5
**Relationship with the patient**	Spouse	40	20
	Father	15	7.5
	Mother	59	29.5
	Sister	18	9
	Brother	15	7.5
	Girl	28	14
	Boy	25	12.5
**The effect of COVID-19 on patient conditions**	Negative effect	157	78.5
	No effect	43	21.5

The mean scores of the quality of life, caregiver burden, and depression were 58.49 ± 6.61, 32.18 ± 11.21, and 11.46 ± 12.49, respectively. Table 2 shows the mean scores of caregiver burden, depression, and quality of life among informal caregivers of the patients undergoing hemodialysis and patients with thalassemia.

**Table 2:** The mean scores of “caregiver burden”, “depression” and “quality of life” in the participants

Variable	Caregiver	Min	Max	Mean	SD
**Caregiver burden**	Hemodialysis patients	5	74	32.46	10.05
	Thalassemia patients	0	55	32.02	11.82
**Depression**	Hemodialysis patients	0	47	15.33	14.28
	Thalassemia patients	0	45	9.38	10.92
**Quality of life**	Hemodialysis patients	39	71	59.61	7.41
	Thalassemia patients	42	70	57.88	6.09

Using stepwise regression, we investigated the predictability of caregiver burden based on depression and quality of life. The results revealed a positive and significant correlation between caregiver burden and the level of depression (*P ≤ 0.0001, r = 0.64*), indicating that the higher the caregiver burden, the higher the level of depression. The coefficient of determination (R2 = 0.32) calculated based on the participants’ depression indicated that this variable predicted 32% of the caregiver’s burden (Table 3).

**Table 3:** Stepwise regression to predict care burden based on depression and quality of life variables in informal caregivers

Predictor variables	β	SD	t	P-value
Constant	33.81	6.05	5.59	> 0.0001
Quality of Life	-1.25	0.1	-1.24	0.21
Depression	0.49	0.05	9.33	> 0.0001

In addition, there was a negative and significant correlation between caregiver burden and quality of life, implying that the lower the quality of life, the higher the caregiver burden (*P = 0.009, r = -0.68)*. There was also a negative correlation between the quality of life and caregiver burden, but this correlation was not statistically significant in the regression model, meaning that the higher the caregiver burden, the lower the quality of life.

The results revealed no significant relationship between age (*P = 0.2*), sex (*P = 0.2*), marital status (*P = 0.16*), and caregiver burden. The mean scores of caregiver burden in married and single men and women were almost the same. One-way analysis of variance (ANOVA) showed no statistically significant difference between caregiver burden and occupation (P = 0.07) as well as between caregiver burden and financial status (*P = 0.12*).

Our results indicated no statistically significant correlation between the level of depression and sex, as well as between the level of depression and marital status (*P < 0.05*), but there was a significant correlation between age and the level of depression, so the level of depression increased with aging (*p < 0.0001*). The ANOVA showed no statistically significant difference in occupation, depression (*P = 0.4*), financial status, and depression among the participants (*P = 0.15*). The results also demonstrated no significant relationship between age and quality of life (p = 0.65), sex and quality of life (*P = 0.7*), and marital status and quality of life (*P = 0.08*). The ANOVA revealed no statistically significant relationship between occupation and quality of life (*P = 0.23*) but a statistically significant relationship was found between financial status and quality of life, indicating that better financial status was associated with a higher level of quality of life (*P = 0.008*).

## Discussion

The present study examined the correlations between caregiver burden, depression, and quality of life among informal caregivers of thalassemia and hemodialysis patients during the COVID-19 pandemic. The results revealed that the level of caregiver burden was moderate in the caregivers of hemodialysis and thalassemia patients. However, Rioux et al. reported a low level of caregiver burden among informal caregivers of patients undergoing hemodialysis [6]. Mollaoğlu et al. and Alnazly et al. found severe levels of caregiver burden in the informal caregivers of patients undergoing hemodialysis [23, 37]. Paramore et al. concluded that the caregiver burden was severe among caregivers of patients with thalassemia [24]. Mashayekhi et al., Abbasi et al., and Cantekin et al. observed that 72.5%, 74.2%, and 86.9% of the informal caregivers experienced moderate to severe levels of caregiver burden [2, 25, 38]. The differences might be due to different instruments used in the studies or because previous studies mostly examined the caregivers of hospitalized patients, but the participants in the preset were not currently hospitalized; Iranian media created adequate training channels for caregivers of chronically ill patients during the COVID-19 pandemic, which covered various medical fields.

Our study showed higher levels of depression among informal caregivers of the patients undergoing hemodialysis than that of patients with thalassemia. Olarte-Durand et al. and Saenz et al. revealed that the COVID-19 pandemic affected public mental health, with several studies showing increased levels of depression [39, 40]. Nadort et al. found that the level of depression in caregivers of the patients’ undergoing hemodialysis was higher before the COVID-19 pandemic [41], while Chávez et al. reported that 86.4% of the caregivers had low depression after the COVID-19 outbreak [42]. Several studies suggested that caregivers of patients have mild to moderate depression [43, 44]. Schneider et al. and Ebadi et al. also reported that caregivers of hemodialysis patients experienced chronic stress and psychological disorders such as anxiety and depression [3, 45]. Therefore, we should pay attention to psychological counseling with a focus on active coping strategies and the improvement of relationships between professional caregivers such as nurses and family caregivers in pandemic conditions.

Based on our findings, the quality of life in the informal caregivers was somewhat desirable, but it was higher in the informal caregivers of patients undergoing hemodialysis than that of patients with thalassemia. Farzi et al. revealed that the mean score of quality of life was low in caregivers of hemodialysis patients [46]. Habibzadeh et al. reported that 52.5% of the informal caregivers of hemodialysis patients had a moderate to low quality of life [47]. The difference between the results of these studies and the current study could be attributed to the instruments used, the level of self-care and other chronic illnesses of patients, sample size, disease severity, and diversity in patients and healthcare centers. Thus, psychologists and psychiatric nurses need to identify and overcome psychological problems, implement training programs, and increase informal caregivers’ knowledge of coping strategies during the pandemic conditions to enhance their quality of life.

The results also showed a statistically significant correlation between caregiver burden and depression, confirming that the higher the depression level, the higher the caregiver burden. Likewise, Tang and Adili et al. reported similar results and highlighted the need to address psychological and physical problems in caregivers [48, 49].

The present study found no statistically significant correlation between caregiver burden and quality of life, which was inconsistent with the studies conducted by Adili et al. and Wicks [49, 50] because most of our participants suffered from thalassemia and their informal caregivers had more adaptation to the disease from the birth of the child and reached a relative stability in their quality of life.

More than half of the caregivers in the present study were mothers. A recent study investigated the burden of caregivers of chronically ill children in Iran and reported that mothers were caregivers in most cases [51]. This study indicated no significant correlation between sex and caregiver burden, as reported by Adili et al. [49], but most studies reported higher caregiver burden among women [52, 53]. Men and women in the current study reported a relatively equal burden of care because men usually had to take care of the other family members in addition to the sick patient.

Two-thirds of the caregivers reported the negative effect of COVID-19 on patient conditions, as confirmed by Arian [54]. Thus, we can argue that informal caregivers and care receivers were both under a lot of pressure during the COVID-19 outbreak because the COVID-19 pandemic disturbed the balance of life and the health of patients.

We observed no significant correlation between age and caregiver burden, as reported by Adili et al., Zahid et al., and Agren et al. [49, 55, 56], but this finding was inconsistent with the results reported by Lee and Rafati et al. [35, 57]. Caregiving roles and responsibilities have involved all family caregivers during the COVID-19 pandemic, which made younger caregivers more distressed because they were less experienced in caring for a patient, and thus underwent more care burden.

The present study found no correlation between caregiver burden and marital and financial status, as evident in other studies (Zahid et al.; Adili et al.) [49, 55], but this finding was inconsistent with the results reported by Hosseini et al. and Perlik et al. [58, 59] because hemodialysis and thalassemia patients need several years of care. Due to the nature of the diseases and the high mortality rate in the COVID-19 pandemic, patient caregivers tried their best to care for patients and complained less about the care burden.

The present study found a significant correlation between age and depression, indicating that the level of depression increased with aging, as reported by Haghighizadeh et al. and Difazio [60, 61]. This is to argue that during the COVID-19 pandemic and lockdown, younger caregivers often tend to spend more time on social media and other news outlets. Thus, increased opportunities for social connection through social media outlets that are readily available to younger caregivers compared to older adults would limit the impact of physical distancing such as depression on them.

Our results suggested no significant correlation between sex and depression, as evident in a study by Haghighizadeh [61], but Ashrafi did not report a similar finding [62]. Depression is usually more common in women, but its equal prevalence in both men and women in this study may indicate that caregiving has a greater negative effect on men [63].

The present study found a relationship between quality of life and caregiver financial status. Since most of the participants in this study were caring for patients with thalassemia, the financial status of the family and informal caregivers could have a significant impact on their quality of life.

This study revealed no significant correlation between caregivers’ age, sex, marital status, and quality of life, as reported by Taqhavi et al. [64]. Given that the quality of life decreases in difficult conditions, such variables cannot be decisive in this regard [65]. Therefore, it is recommended to teach problem-solving skills to informal caregivers to improve their quality of life.

### Limitations

The present study also had several limitations. This study was conducted only in one medical center, so we could not consider the effect of the quality of services in different centers and the severity of disorders on the caregiver burden. We only focused on informal caregivers of thalassemia and hemodialysis patients, while investigating caregiver burden, quality of life, and depression in informal caregivers of patients with diabetes, chronic obstructive pulmonary diseases, mental disorders, Alzheimer’s disease, and cancers could also provide rich information. Another limitation was the use of self-report instruments for data collection, which was not a gold standard method for measuring variables.

We recommend that further studies assess factors affecting caregivers’ burden and identify the effectiveness of nursing and psychological interventions in improving the burden, depression, and quality of life of caregivers of hemodialysis and thalassemia patients.

### Practical, educational, and research implications

Our findings highlighted the need for greater efforts to decrease caregiver burden and depression and promote the quality of life of caregivers of thalassemia and hemodialysis patients in Iran. Nursing managers must be aware of nurses’ knowledge, required recourses to meet caregivers’ needs, and motivating strategies to promote caregiver self-confidence when caring for chronic patients such as thalassemia and hemodialysis in new situations. This study provided evidence-based insights into organizational strategic planning and can help to identify opportunities and threats of caregivers’ burden in new situations. The results will also be useful for nursing managers, policy- and decision-makers, and nursing educators to develop effective strategies to promote the quality of nursing care, implement home nursing care, and reduce or eliminate challenges in this group of patients. These strategies may include caregivers’ needs assessment, modification of service delivery to this group, creativity in different aspects of the educational curricula, and comprehensive training programs for care providers in crises.

## Conclusion

The results showed the negative impacts of the COVID-19 pandemic on informal caregivers of patients. Iranian informal caregivers of chronically ill patients, especially those caring for hemodialysis and thalassemia patients experienced moderate levels of the burden of care, depression, and low quality of life. Therefore, Iranian healthcare officials must take necessary measures to reduce the burden of care and depression and improve the quality of life of informal caregivers.

## Data Availability

The datasets generated and/or analyzed during the current study are not publicly available due to keeping participants’ information confidential but are available from the corresponding author at reasonable request.
